# Rates of adverse clinical events in patients with chronic kidney disease: analysis of electronic health records from the UK clinical practice research datalink linked to hospital data

**DOI:** 10.1186/s12882-023-03119-z

**Published:** 2023-04-05

**Authors:** Dustin J. Little, Matthew Arnold, Katarina Hedman, Ping Sun, Syed Asif Haque, Glen James

**Affiliations:** 1 Late Cardiovascular, Renal, Metabolism, BioPharmaceuticals R&D, AstraZeneca, Gaithersburg, MD 20876 USA; 2 Real World Data Science, BioPharmaceuticals Medical, AstraZeneca, Cambridge, UK; 3 Biometrics CVRM, BioPharmaceuticals R&D, AstraZeneca, Gothenburg, Sweden; 4 Real World Data Science, Oncology Business Unit, AstraZeneca, Cambridge, UK; 5 Global Patient Safety BioPharma, BioPharmaceuticals Medical, AstraZeneca, Gaithersburg, MD USA; 6 Cardiovascular, Renal, Metabolism Epidemiology, BioPharmaceuticals Medical, AstraZeneca, Cambridge, UK; 7 Present Address: Integrated Evidence Generation & Business Innovation, Bayer PLC, Reading, UK

**Keywords:** Chronic kidney disease, Hemodialysis, Hyperkalemia, Pneumonia, Sepsis

## Abstract

**Background:**

Further understanding of adverse clinical event rates in patients with chronic kidney disease (CKD) is required for improved quality of care. This study described baseline characteristics, adverse clinical event rates, and mortality risk in patients with CKD, accounting for CKD stage and dialysis status.

**Methods:**

This retrospective, noninterventional cohort study included data from adults (aged ≥ 18 years) with two consecutive estimated glomerular filtration rates of < 60 ml/min/1.73 m^2^, recorded ≥ 3 months apart, from the UK Clinical Practice Research Datalink of electronic health records obtained between January 1, 2004, and December 31, 2017. Select adverse clinical events, associated with CKD and difficult to quantify in randomized trials, were assessed; defined by Read codes and International Classification of Diseases, Tenth Revision codes. Clinical event rates were assessed by dialysis status (dialysis-dependent [DD], incident dialysis-dependent [IDD], or non–dialysis-dependent [NDD]), dialysis modality (hemodialysis [HD] or peritoneal dialysis [PD]), baseline NDD-CKD stage (3a–5), and observation period.

**Results:**

Overall, 310,953 patients with CKD were included. Comorbidities were more common in patients receiving dialysis than in NDD-CKD, and increased with advancing CKD stage. Rates of adverse clinical events, particularly hyperkalemia and infection/sepsis, also increased with advancing CKD stage and were higher in patients on HD versus PD. Mortality risk during follow-up (1–5-year range) was lowest in patients with stage 3a NDD-CKD (2.0–18.5%) and highest in patients with IDD-CKD (26.3–58.4%).

**Conclusions:**

These findings highlight the need to monitor patients with CKD for comorbidities and complications, as well as signs or symptoms of clinical adverse events.

**Supplementary Information:**

The online version contains supplementary material available at 10.1186/s12882-023-03119-z.

## Background

Chronic kidney disease (CKD) is prevalent in individuals with diabetes, hypertension, and obesity [1]. Compared with the general population, CKD is associated with an increased risk of cardiovascular disease, decreased health-related quality of life, premature mortality, and complications, such as anemia and metabolic bone disease [1–4].

Robust, well-designed clinical trials are less common within nephrology compared with other specialties [5]. Furthermore, patients with CKD are more likely to be excluded from randomized controlled trials of cardiovascular interventions than those without CKD; a systematic literature review of 371 trials found that 57.1% of trials excluded patients with CKD [6]. Limited knowledge of adverse clinical event rates in patients with CKD can hinder quality of care assessments and patient counseling and education, as well as impair the development and delivery of new therapies [7]. Further understanding of clinical event rates is required to improve quality of care.

This study aimed to describe baseline characteristics and rates of adverse clinical events and mortality in UK patients with CKD, using data from the UK Clinical Practice Research Datalink (CPRD) linked to hospital data and mortality statistics. The impact of disease stage, dialysis status, and dialysis modality on adverse clinical event rates and mortality was also assessed.

## Methods

### Study design and data source

This retrospective, noninterventional cohort study used data from the UK CPRD (ISAC protocol number, 19_172) between January 1, 2004, and December 31, 2017. The CPRD is a database of anonymized electronic medical records from UK primary care practices, covering ~ 16 million patients [8]. Patients in the CPRD are broadly representative of the UK general population in terms of age, sex, and ethnicity [9]. Data were linked to Hospital Episode Statistics (HES) [10], a secondary care database in England, and the Office for National Statistics (ONS) mortality file, a death registry for England and Wales [11]. All data were anonymized at the point of extraction, and no personally identifiable information was available.

### Study population

Eligible patients were aged ≥ 18 years and had a record of CKD between January 1, 2004, and December 31, 2017. CKD was identified based on two consecutive estimated glomerular filtration rates (eGFRs) of < 60 ml/min/1.73 m^2^, recorded ≥ 3 months apart. Patients with a history of kidney transplantation, or those who died within the first 6 months after the index date, were excluded.

Dialysis status was identified using Office of Population Censuses and Surveys fourth revision procedure codes for kidney replacement therapy. eGFRs were calculated from serum creatinine values, using the Chronic Kidney Disease Epidemiology Collaboration equation, which includes modifiers for age, sex, and race [12]. Non–dialysis-dependent (NDD)-CKD stage was defined as follows: stage 3a, 45–59 ml/min/1.73 m^2^; stage 3b, 30–44 ml/min/1.73 m^2^; stage 4, 15–29 ml/min/1.73 m^2^; and stage 5, < 15 ml/min/1.73 m^2^. Incident dialysis-dependent (IDD)-CKD was defined as the first code for dialysis during follow-up, with no dialysis code recorded in a 12-month lookback period. Chronic dialysis was assumed from the time of initial code recording through end of follow-up. For these patients, we assessed adverse clinical events within the first 3–6 months following the first dialysis code. It was possible for patients who started the study with NDD-CKD to have begun dialysis during the study and transitioned to the IDD-CKD cohort. The index date in the NDD-CKD cohort was defined as the date of the second eGFR measurement. In the IDD-CKD cohort, this was the date of dialysis, which occurred after the NDD-CKD index date.

### Study outcomes

The primary outcomes were baseline characteristics (defined as the time of study inclusion), adverse clinical event rates, and mortality risk. Baseline characteristics and pre-specified adverse clinical events were extracted using both primary and secondary care data. Laboratory covariates were obtained from the single value obtained closest to the date of study inclusion, at or up to 12 months prior to inclusion. Comorbidity data were obtained using diagnostic codes identified in the 24 months prior to inclusion. Medication data were obtained from prescription-only medications that were active or prescribed within 90 days prior to inclusion.

Adverse clinical event rates were assessed for all patients using general practice, and inpatient and outpatient data, and were reported by dialysis status (dialysis-dependent [DD]-CKD, IDD-CKD, or NDD-CKD), dialysis modality (hemodialysis [HD] or peritoneal dialysis [PD]), baseline NDD-CKD stage (all at index), and observation period (2004–2008, 2009–2014, and 2015–2017). Events were defined by Read codes and International Classification of Diseases, Tenth Revision codes (Table S1). The following events of interest were assessed as they may be difficult to quantify accurately in randomized trials: infection/sepsis, urinary tract infection, gastrointestinal hemorrhage, hypoglycemia, pancreatitis, acidosis, hyperkalemia, rhabdomyolysis, severe cutaneous adverse reactions, pure red cell aplasia, tachycardia, thyroid disorders (hypo/hyperthyroidism), pneumonia/respiratory infection, hepatic disorders, seizure, retinal disorders, and allergic and anaphylaxis events.

### Statistical analysis

The study was purely descriptive without formal hypothesis testing or comparisons. Summary statistics included the number of patients, mean, standard deviation, median, 25th and 75th percentile values, and proportions of patients with missing data in covariates. Incidence rates per 100 person-years (PY) were calculated as the number of events that occurred during patient follow-up time at risk (time until the first event or last available follow-up, whichever came first).

All-event rates were calculated as the total number of events (irrespective of whether some patients provided multiple events occurring on different days) that occurred during patient follow-up time at risk (years) until patients’ last available follow-up.

Kaplan–Meier (KM) risk curves were used to describe time to mortality and to estimate 1-, 2-, 3-, and 5-year KM risk (%), including estimated 95% confidence intervals (CIs).

## Results

### Baseline characteristics

**Fig. 1:**
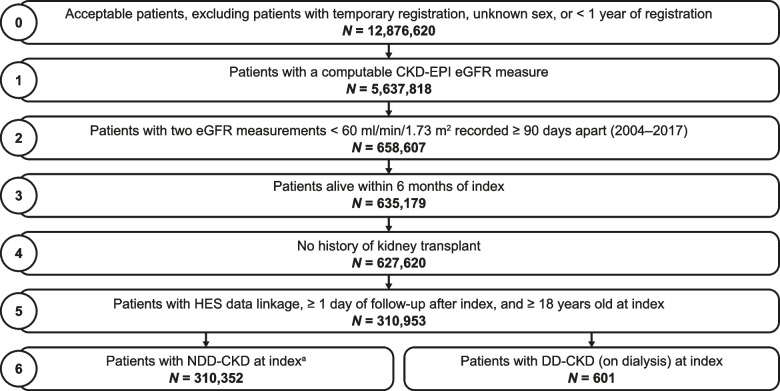
Flow diagram of patient selection from CPRD. ^a^Includes patients in the IDD cohort who were in the NDD cohort at index CKD, chronic kidney disease; CKD-EPI, Chronic Kidney Disease Epidemiology Collaboration; CPRD, Clinical Practice Research Datalink; DD, dialysis-dependent; eGFR, estimated glomerular filtration rate; HES, Hospital Episode Statistics; IDD, incident dialysis-dependent; NDD, non–dialysis-dependent

Overall, 310,953 patients with CKD were identified (a prevalence of 2.4% in the data source); at index, 0.2% (601) and 99.8% (310,352) had DD-CKD and NDD-CKD, respectively (Fig. 1). Among patients with NDD-CKD, 71.7% (222,651), 23.0% (71,367), 4.8% (15,033), and 0.4% (1,301) were categorized as stage 3a, stage 3b, stage 4, and stage 5, respectively. Some patients who started the study in the NDD-CKD cohort entered the DD-CKD cohort (and, by definition, the IDD-CKD subcohort) during follow-up. Baseline characteristics were assessed in all patients who met the criteria for the NDD-CKD, IDD-CKD, and DD-CKD cohorts at index or during follow-up, resulting in patient overlap between the DD-CKD and IDD-CKD cohorts (Table 1).

**Table 1:** Baseline demographics and disease characteristics of patients with CKD by dialysis status and disease stage

Demographic	DD-CKD^a^	IDD-CKD^a^	NDD-CKD				
	**(*n* = 3,931)**	**(*n* = 3,330)**	**Overall**	**Stage 3a**	**Stage 3b**	**Stage 4**	**Stage 5**
			**(*n* = 310,352)**	**(*n* = 222,651)**	**(*n* = 71,367)**	**(*n* = 15,033)**	**(*n* = 1,301)**
Age, years, median (IQR)	67 (57–74)	67 (57–74)	76 (69–83)	75 (68–81)	80 (74–85)	82 (74–87)	78 (68–85)
Female, *n* (%)	1,539 (39.2)	1,301 (39.1)	187,419 (60.4)	131,817 (59.2)	45,676 (64.0)	9,212 (61.3)	714 (54.9)
Race, *n* (%)							
White	3,540 (90.1)	3,016 (90.6)	291,979 (94.1)	209,584 (94.1)	67,226 (94.2)	13,983 (93.0)	1,186 (91.2)
Black	111 (2.8)	86 (2.6)	1,915 (0.6)	1,393 (0.6)	361 (0.5)	139 (0.9)	22 (1.7)
Asian	199 (5.6)	161 (5.3)	4,572 (1.5)	3,363 (1.5)	932 (1.3)	245 (1.8)	32 (2.9)
Other	42 (1.1)	34 (1.0)	1,838 (0.6)	1,340 (0.6)	399 (0.6)	83 (0.6)	16 (1.2)
Unknown	18 (0.5)	18 (0.5)	9,422 (3.0)	6,498 (2.9)	2,333 (3.3)	552 (3.7)	39 (3.0)
BMI, kg/m^2^, median (IQR)	27.1 (23.8–31.5)	27.1 (23.8–31.5)	26.9 (23.9–30.4)	27.0 (24.1–30.4)	26.6 (23.5–30.1)	26.5 (23.4–30.2)	26.1 (23.0–30.2)
Systolic BP, mmHg, median (IQR)	138 (124–150)	138 (124–150)	140 (129–150)	140 (129–149)	140 (128–150)	140 (125–150)	140 (125–150)
Diastolic BP, mmHg, median (IQR)	74 (66–80)	74 (66–80)	78 (70–82)	78 (70–83)	77 (70–82)	75 (68–80)	75 (68–81)
Follow-up duration, years, median (IQR)	2.3 (0.7–4.8)	1.9 (0.5–4.1)	4.9 (2.4–8.1)	5.2 (2.6–8.3)	4.4 (2.2–7.5)	3.3 (1.6–6.0)	2.7 (1.4–5.2)
Medical history and comorbidities^b^, *n* (%)							
Angina pectoris	470 (12.0)	413 (12.4)	21,598 (7.0)	14,192 (6.4)	5,833 (8.2)	1,461 (9.7)	112 (8.6)
Coronary artery disease	761 (19.4)	673 (20.2)	16,646 (5.4)	11,186 (5.0)	4,209 (5.9)	1,137 (7.6)	114 (8.8)
Myocardial infarction	322 (8.2)	270 (8.1)	8,573 (2.8)	5,130 (2.3)	2,561 (3.6)	807 (5.4)	75 (5.8)
Atrial fibrillation	248 (6.3)	216 (6.5)	15,469 (5.0)	9,957 (4.5)	4,452 (6.2)	988 (6.6)	72 (5.5)
Atrial flutter	780 (19.8)	680 (20.4)	22,751 (7.3)	14,017 (6.3)	6,801 (9.5)	1,786 (11.9)	147 (11.3)
Diabetes (type 1 or 2)	1,577 (40.1)	1,374 (41.3)	40,667 (13.1)	27,328 (12.3)	10,260 (14.4)	2,798 (18.6)	281 (21.6)
Diabetic nephropathy	660 (16.8)	594 (17.8)	1,374 (0.4)	686 (0.3)	370 (0.5)	245 (1.6)	73 (5.6)
Cardiac valve disease	576 (14.7)	501 (15.0)	8,495 (2.7)	5,321 (2.4)	2,480 (3.5)	643 (4.3)	51 (3.9)
Dyslipidemia	855 (21.8)	759 (22.8)	15,470 (5.0)	10,661 (4.8)	3,763 (5.3)	950 (6.3)	96 (7.4)
Hypertension	2,375 (60.4)	2,049 (61.5)	99,588 (32.1)	67,787 (30.4)	25,345 (35.5)	5,883 (39.1)	573 (44.0)
Peripheral artery disease	683 (17.4)	593 (17.8)	10,696 (3.4)	6,588 (3.0)	3,117 (4.4)	895 (6.0)	96 (7.4)
Thrombosis	423 (10.8)	359 (10.8)	8,257 (2.7)	5,536 (2.5)	2,165 (3.0)	513 (3.4)	43 (3.3)
Laboratory parameters, median (IQR)							
Hb, g/dL	10.6 (9.5–11.9)	10.6 (9.5–11.9)	13.3 (12.2–14.3)	13.5 (12.5–14.4)	12.8 (11.7–13.8)	11.9 (10.7–13.0)	11.0 (9.8–12.2)
CRP, mg/L	10.0 (5.0–34.0)	10.0 (4.5–32.0)	5.0 (3.0–11.0)	5.0 (2.6–10.0)	6.0 (3.0–14.0)	8.0 (4.0–19.2)	10.0 (4.4–30.0)
UACR, mg/g^c^	20.4 (3.9–113.3)	21.2 (4.3–122.6)	1.8 (0.8–4.4)	1.5 (0.7–3.4)	2.1 (1.0–6.7)	5.2 (1.9–17.3)	15.7 (2.8–68.8)
Medications, *n* (%)							
ACEi	1,160 (29.5)	1,007 (30.2)	104,463 (33.7)	71,933 (32.3)	26,426 (37.0)	5,695 (37.9)	409 (31.4)
ARB	829 (21.1)	758 (22.8)	41,319 (13.3)	28,670 (12.9)	10,054 (14.1)	2,412 (16.0)	183 (14.1)
Statins	2,183 (55.5)	1,961 (58.9)	122,784 (39.6)	90,009 (40.4)	26,478 (37.1)	5,767 (38.4)	530 (40.7)
NSAID	1,583 (40.3)	1,409 (42.3)	134,511 (43.3)	93,524 (42.0)	33,465 (46.9)	7,019 (46.7)	503 (38.7)
Loop diuretics	1,874 (47.7)	1,723 (51.7)	58,086 (18.7)	31,645 (14.2)	19,397 (27.2)	6,474 (43.1)	570 (43.8)

*Abbreviations: ACEi* angiotensin-converting enzyme inhibitor, *ARB* angiotensin receptor blocker, *BMI* body mass index, *BP* blood pressure, *CKD* chronic kidney disease, *CRP* C-reactive protein, *DD* dialysis-dependent, *Hb* hemoglobin, *IDD* incident dialysis-dependent, *IQR* interquartile range, *NDD* non–dialysis-dependent, *NSAID* non-steroidal anti-inflammatory drug, *UACR* urine albumin-to-creatinine ratio

^a^Includes patients who had NDD-CKD at index

^b^Comorbidities with > 5.0% prevalence in any cohort

^c^Proportions of patients with missing data were 86.1% (DD-CKD), 84.8% (IDD-CKD), 91.9% (NDD-CKD overall), 91.8% (stage 3a NDD-CKD), 92.4% (stage 3b NDD-CKD), 91.5% (stage 4 NDD-CKD), and 92.9% (stage 5 NDD-CKD)

Patients with NDD-CKD were older, with a median (interquartile range [IQR]) age of 76 (69–83) years, compared with 67 (57–74) years for patients with DD-CKD and IDD-CKD (Table 1). Overall, 60.4%, 39.2%, and 39.1% of patients with NDD-CKD, DD-CKD, and IDD-CKD, respectively, were female (Table 1). The most common comorbidities at baseline were hypertension (32.1%, 60.4%, and 61.5%), and diabetes (13.1%, 40.1%, and 41.3%) for patients with NDD-CKD, DD-CKD, and IDD-CKD, respectively. Comorbidities were less common, median hemoglobin values were higher, and C-reactive protein values were lower in patients with NDD-CKD compared with DD-CKD or IDD-CKD (Table 1). Among patients with NDD-CKD, the prevalence of comorbidities generally increased with decreasing eGFR (Table 1). Median (IQR) follow-up duration was 27.5 (8.8–57.0) months for DD-CKD, compared with 22.7 (6.4–49.0) and 59.1 (29.2–96.6) months for IDD-CKD and NDD-CKD, respectively (Table 1). A larger proportion of patients with NDD-CKD were alive at last assessment compared with DD-CKD and IDD-CKD (44.9%, 30.4%, and 28.6%, respectively). More patients with stage 3a NDD-CKD were alive at last assessment (52.4%), compared with stage 3b (27.7%), stage 4 (16.8%), or stage 5 (19.3%) NDD-CKD.

### Adverse clinical events

**Fig. 2:**
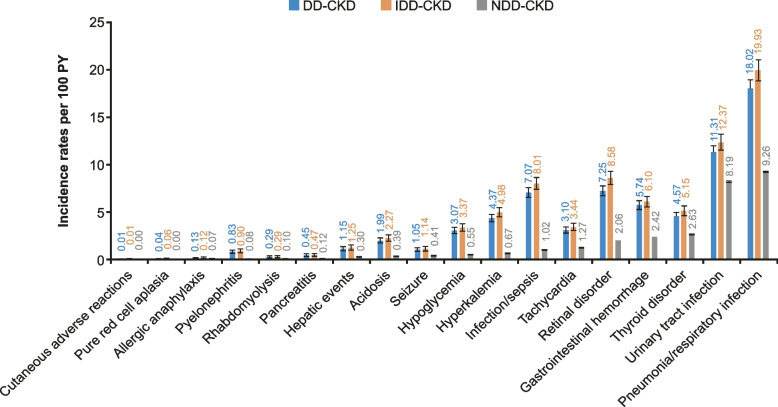
Incidence rates per 100 PY (95% CI) for patients with CKD by dialysis status CI, confidence interval; CKD, chronic kidney disease; DD, dialysis-dependent; IDD, incident dialysis-dependent; NDD, non–dialysis-dependent; PY, person-years

The most common adverse clinical events per 100 PY among patients with DD-CKD and IDD-CKD included pneumonia/respiratory infection (incidence rate [95% CI] 18.0 [17.2–18.9] and 19.9 [18.9–21.1], respectively), urinary tract infection (11.3 [10.7–12.0] and 12.4 [11.6–13.2]), infection/sepsis (7.1 [6.6–7.6] and 8.0 [7.4–8.7]), and seizure (1.05 [0.87–1.25] and 1.14 [0.93–1.38]) for DD-CKD and IDD-CKD, respectively (Fig. 2). The most common adverse clinical events per 100 PY among patients with NDD-CKD included pneumonia/respiratory infection (incidence rate [95% CI] 9.3 (9.2–9.3) and urinary tract infection (8.2 [8.2–8.2]) (Fig. 2). All-event rates followed similar patterns and are shown in Fig. S1. Overall, incidence rates of adverse events were highest during the more recent observation periods for DD-CKD and IDD-CKD; among patients with NDD-CKD, incidence rates of all adverse events were highest in 2009–2014 and lowest in 2004–2008 (Fig. S2).

**Fig. 3:**
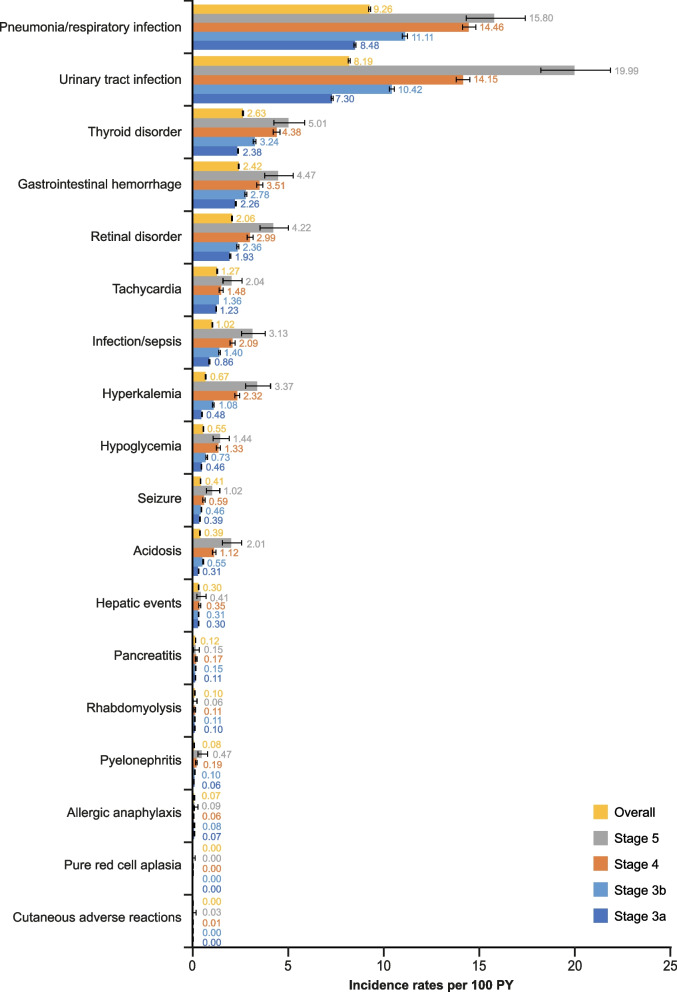
Incidence rates per 100 PY (95% CI) for patients with NDD-CKD by disease stage CI, confidence interval; CKD, chronic kidney disease; NDD, non–dialysis-dependent; PY, person-years

Incidence rates and all-event rates per 100 PY were generally higher in patients receiving dialysis compared with those who were not (Fig. 2; Fig. S1). Incidence rates of hyperkalemia and infection/sepsis were ~ 6.5-fold and 6.9-fold higher, respectively, in patients with DD-CKD compared with patients with NDD-CKD. In patients with IDD-CKD, incidence rates were ~ 7.4-fold and 7.9-fold higher versus NDD-CKD for hyperkalemia and infection/sepsis, respectively. The difference between groups was less pronounced for other events; for example, the incidence rate of seizures was ~ threefold higher in patients on dialysis versus NDD-CKD. For patients with NDD-CKD, incidence and all-event rates per 100 PY were generally higher with increasing CKD stage. Rates were higher in patients with stage 4 or stage 5 NDD-CKD compared with stages 3a and 3b NDD-CKD (Fig. 3; Fig. S3). Incidence rates were higher in patients with DD-CKD and IDD-CKD on HD versus PD, particularly in the most common adverse events (pneumonia/respiratory infection and urinary tract infection; Fig. S4).

### Mortality

Risk of mortality during follow-up (1–5-year range) was lower in patients with stage 3a NDD-CKD (2.0–18.5%) and stage 3b NDD-CKD (4.0–33.7%) than in patients with stage 4 NDD-CKD (7.4–51.2%), stage 5 NDD-CKD (10.2–58.3%), DD-CKD (22.5–52.5%), or IDD-CKD (26.3–58.4%) (Table 2). This trend continued throughout follow-up beyond year 5 (Fig. S5). In patients on dialysis, risk of mortality during follow-up (1–5-year range) was lower in patients on PD (11.76–46.43%) compared with HD (24.09–61.94%), and in patients whose dialysis modality was not specified (30.91–59.95%; Table S2).

**Table 2:** Patient mortality and risk by dialysis status

Patient mortality and risk		DD-CKD	IDD-CKD	NDD-CKD				
		**Overall** **(*n* = 3,931)**	**Overall** **(*n* = 3,330)**	**Overall** **(*n* = 310,352)**	**Stage 3a** **(*n* = 222,651)**	**Stage 3b** **(*n* = 71,367)**	**Stage 4** **(*n* = 15,033)**	**Stage 5** **(*n* = 1,301)**
1 year	Events, *n*	857	845	8,298	4,338	2,766	1,069	125
	Mortality risk, KM% (95% CI)	22.47 (21.13–23.79)	26.29 (24.74–27.80)	2.79 (2.73–2.85)	2.04 (1.98–2.10)	4.03 (3.89–4.18)	7.43 (7.00–7.85)	10.21 (8.49–11.89)
2 years	Events, *n*	299	277	15,517	8,145	5,281	1,890	201
	Mortality risk, KM% (95% CI)	31.46 (29.93–32.96)	36.44 (34.69–38.14)	8.30 (8.20–8.40)	6.08 (5.97–6.18)	12.14 (11.89–12.38)	21.32 (20.64–22.00)	27.64 (25.03–30.16)
3 years	Events, *n*	218	186	13,964	7,661	4,663	1,501	139
	Mortality risk, KM% (95% CI)	38.97 (37.30–40.59)	44.41 (42.53–46.23)	13.69 (13.56–13.82)	10.21 (10.08–10.35)	19.84 (19.53–20.15)	33.28 (32.47–34.08)	40.79 (37.83–43.60)
5 years	Events, *n*	316	255	22,701	13,221	7,348	1,968	164
	Mortality risk, KM% (95% CI)	52.51 (50.62–54.32)	58.36 (56.26–60.36)	23.80 (23.63–23.96)	18.50 (18.32–18.68)	33.68 (33.29–34.06)	51.18 (50.27–52.07)	58.30 (55.14–61.23)

Where KM% is KM risk function multiplied by 100, including estimated 95% CI

*Abbreviations: CI* confidence interval, *CKD* chronic kidney disease, *DD* dialysis-dependent, *IDD* incident dialysis-dependent, *KM* Kaplan–Meier, *NDD* non–dialysis-dependent

## Discussion

This large retrospective analysis from UK primary care linked to hospital data examined baseline characteristics, adverse clinical events, and mortality in patients with DD-CKD, IDD-CKD, and NDD-CKD. Patients with advanced CKD (stages 4 and 5) had more comorbidities at baseline, and a higher risk of adverse clinical events and mortality, than patients with stages 3a and 3b NDD-CKD.

Baseline characteristics of patients with CKD in this study were generally consistent with previous studies, including an analysis of adverse clinical events in patients with CKD from the US TriNetX database (*n* = 492,141) [7] and other large cohort studies [13, 14]. The most common baseline comorbidities among patients with CKD in the present study were hypertension and diabetes, similar to previous findings [13, 14]. Comorbidities were more common in patients receiving dialysis than in patients with NDD-CKD, and increased in prevalence with advancing CKD stage.

Rates of adverse clinical events were generally higher in patients receiving dialysis, particularly in those starting dialysis during follow-up, compared with NDD-CKD. This difference was most notable for incidence rates of hyperkalemia and infection/sepsis, which were up to eightfold higher in patients receiving dialysis. Both hyperkalemia and infections are common in patients undergoing dialysis [15, 16]. Hyperkalemia is a consequence of severely reduced potassium excretion in patients with reduced eGFR [17], while patients receiving HD are at increased risk of acquiring nosocomial infections due to weakened immune systems, frequent catheterization, and increased hospitalizations [18, 19]. Treating these adverse events incurs substantial healthcare costs and resource utilization [18, 19]. Additionally, the incidence of seizure was almost three times higher in patients on dialysis compared with NDD-CKD. This concurs with previous data showing that neurological disorders are common in patients with CKD and may increase in frequency with advancing disease [20]. Collectively, the increased incidence of adverse clinical events likely reflects the higher comorbidity burden of patients on or transitioning to dialysis versus those with NDD-CKD.

Our findings showed a temporal trend in adverse events among patients with DD-CKD and IDD-CKD, whereby incidence rates were highest in the most recent observation periods. Conversely, incidence rates of adverse events were highest in 2009–2014 among patients with NDD-CKD. These findings could reflect increased monitoring and improvements in clinical coding, electronic health records, and adverse event reporting in patients receiving dialysis. They may also be attributable to changes in risk characteristics among patients on dialysis over time; further research is required to investigate the causative factors. We observed increased incidence rates of adverse events, particularly infection, in patients on HD compared with PD. This finding may reflect the adverse events included in this category, as data from a previous study reported similar overall infection rates in patients on HD and PD, but with variation in the type of infection and risk over time [21]. In another study, pneumonia risk was higher in patients on HD versus PD [22].

Incidence and all-event rates of adverse clinical events generally increased with decreasing eGFR. This aligns with previous findings from the US TriNetX network, which observed notable increases in incidence rates for hyperkalemia, acidosis, and sepsis between patients with stage 3 and stage 5 NDD-CKD [7]. Factors associated with hyperkalemia in patients with CKD, in addition to reduced kidney function, include concomitant medication use (e.g., renin–angiotensin–aldosterone system inhibitors and potassium-sparing diuretics), and comorbidities (e.g., diabetes and cardiovascular disease) [23, 24]. In CKD, metabolic acidosis often develops when eGFR decreases to values consistent with CKD stage 4, due to factors such as impaired ammonia excretion, reduced tubular bicarbonate reabsorption, and insufficient renal bicarbonate production [25]. Our findings are consistent with studies that have shown an increased risk of infection and sepsis with declining eGFR in patients with NDD-CKD [26, 27].

The increased rates of retinal disorders and hypoglycemia with decreasing eGFR in our study were consistent with established links between CKD, diabetes, and retinopathy [28–32]. CKD and retinopathy share common vascular risk factors, including diabetes, hypertension, smoking, and obesity [33]. Presence of CKD adds a risk factor for developing hypoglycemia through altered drug metabolism, malnutrition, infections, problems linked to dialysis, associated cardiac and hepatic disease, and impaired renal glucose release [31].

Our findings also showed a clear increase in mortality risk with increasing CKD severity. Patients with stage 4 or stage 5 NDD-CKD, IDD-CKD, or DD-CKD had 5-year mortality risks as high as 51.2–58.4%, compared with 18.5–33.7% in patients with stages 3a and 3b NDD-CKD. The highest risk of mortality at 5-year follow-up was observed for patients with IDD-CKD (58.4%). This aligns with previous studies showing high mortality within the first weeks of initiating HD, possibly due to elevated cardiovascular event rates after dialysis initiation [34, 35]. When patients were stratified according to dialysis modality, mortality risk was lower among patients on PD compared with HD. Few studies have directly compared mortality rates between HD and PD, and current evidence is contradictory, with mortality rates potentially varying according to patient characteristics [36–38].

The large increase in mortality risk between patients with stages 3a and 3b NDD-CKD and those on dialysis may be a consequence of the increase in comorbidity burden and adverse clinical events as CKD progresses, as demonstrated by our findings. Collectively, our data highlight the need to monitor patients in accordance with clinical guideline recommendations [39, 40] to promote early diagnosis of comorbidities, such as cardiovascular disease and diabetes, which can increase the risk of adverse clinical events and mortality [41–43].

Strengths of this study include the large sample size, with longitudinal assessment of a contemporary and representative cohort of patients with CKD from the UK, and advantages inherent to the CPRD, including linkage to secondary care data and ONS mortality records, comprehensive reporting of laboratory tests and adverse clinical events, and the granularity of the coding in the CPRD. Moreover, a large number of adverse events were assessed, many of which are relatively uncommon and are not frequently assessed in randomized clinical trials in nephrology.

This study was limited by a lack of formal statistical comparisons between groups. Ongoing studies, such as DISCOVER CKD (ClinicalTrials.gov identifier: NCT04034992), a hybrid, multinational observational cohort study in patients with CKD [44], will help to address this knowledge gap. It is also possible that undiagnosed or asymptomatic patients with CKD were missed. However, CKD prevalence in CPRD is comparable to that reported by the Health Survey for England, suggesting that most patients with CKD are captured in CPRD [45].

CPRD data are collected as part of routine clinical practice and are not specifically collected or intended for research purposes; as such, the present findings depend on the quality and completeness of the data recorded. For example, our analysis of comorbidity prevalence required the assumption that no record of a disease meant that a patient did not have that disease, which may not have been accurate in all cases. Underreporting or misclassification of clinical coding and outcomes could have also led to underestimation of comorbidities and incident events. Conversely, all-event rates require cautious interpretation as each occurrence of an outcome may generate more than one entry/coding in the patients’ medical records, which could cause all-event rates to be overestimated. The categorization of patients according to CKD-associated risk was defined using eGFR thresholds, as opposed to both eGFR and albuminuria as is recommended by KDIGO 2012 guidelines [40]. Finally, data specific to dialysis may be lacking since CPRD and HES are not the main sources of clinical recording for these patients. Patients included in CPRD are broadly representative of the UK population [9], but generalization of the study results to other countries may be limited by differences between patient populations.

## Conclusions

In this retrospective, longitudinal analysis, comorbidities, adverse clinical event rates, and mortality risk were higher in patients with stage 4 or 5 NDD-CKD, DD-CKD, and particularly among those with IDD-CKD, compared with stages 3a and 3b NDD-CKD. Adverse event rates and mortality were also higher in patients on HD compared with PD.

Our findings highlight the need to monitor patients with CKD for comorbidities and complications, as well as signs or symptoms of clinical adverse events, such as hyperkalemia, hypoglycemia, retinal disorders, seizures, and infection/sepsis. These findings will also help government agencies and payers to optimize delivery of innovative therapies to patients and physicians, and help improve clinical management of patients with CKD.

## Supplementary Information

**Additional file 1: Table S1.** Read codes and ICD-10 codes for clinical events of interest. **Table S2.** Patient mortality and risk by dialysis status and modality. **Fig. S1.** All-event rates per 100 PY (95% CI) for patients with CKD by dialysis status. **Fig. S2.** Incidence rates of adverse clinical events per 100 PY (95% CI) for patients with CKD over time. **Fig. S3.** All-event rates per 100 PY (95% CI) for patients with NDD-CKD by disease stage. **Fig. S4.** Incidence rates per 100 PY (95% CI) for patients with DD-CKD or IDD-CKD by dialysis modality. **Fig. S5.** Risk of mortality for patients with NDD-CKD by stage, IDD-CKD, and DD-CKD.

## Data Availability

Data underlying the findings described in this manuscript may be obtained in accordance with AstraZeneca’s data sharing policy described at https://astrazenecagroup-dt.pharmacm.com/DT/Home, or from Dustin Little (Dustin.little@astrazeneca.com), upon reasonable request.

## Abbreviations

ACEi: Angiotensin-converting enzyme inhibitor
ARB: Angiotensin receptor blocker
BMI: Body mass index
BP: Blood pressure
CIs: Confidence intervals
CKD: Chronic kidney disease
CKD EPI: Chronic Kidney Disease Epidemiology Collaboration
CRP: C-reactive protein
CPRD: Clinical Practice Research Datalink
DD: Dialysis-dependent
eGFR: Estimated glomerular filtration rate
HD: Hemodialysis
HES: Hospital Episode Statistics
Hb: Hemoglobin
IDD: Incident dialysis-dependent
IQR: Interquartile range
KM: Kaplan–Meier
NDD: Non–dialysis dependent
NSAID: Non-steroidal anti-inflammatory drug
ONS: Office for National Statistics
PD: Peritoneal dialysis
PY: Person-years
UACR: Urine albumin-to-creatinine ratio
