# Supplementary material for: Rates of adverse clinical events in patients with chronic kidney disease: analysis of electronic health records from the UK clinical practice research datalink linked to hospital data
Source: BMC Nephrol. 2023 Apr 5;24:91. doi: 10.1186/s12882-023-03119-z (PMC10077632; doi:10.1186/s12882-023-03119-z)
Supplement: Supplementary file 1 — Additional file 1: Table S1. Read codes and ICD-10 codes for clinical events of interest. Table S2. Patient mortality and risk by dialysis status and modality. Fig. S1. All-event rates per 100 PY (95% CI) for patients with CKD by dialysis status. Fig. S2. Incidence rates of adverse clinical events per 100 PY (95% CI) for patients with CKD over time. Fig. S3. All-event rates per 100 PY (95% CI) for patients with NDD-CKD by disease stage. Fig. S4. Incidence rates per 100 PY (95% CI) for patients with DD-CKD or IDD-CKD by dialysis modality. Fig. S5. Risk of mortality for patients with NDD-CKD by stage, IDD-CKD, and DD-CKD. [file 12882_2023_3119_MOESM1_ESM.doc]

Table S1 Read codes and ICD-10 codes for clinical events of interest

| **Outcome** | **Read codes** | **ICD-10 codes** |
| --- | --- | --- |
| Infection/sepsis events | A545.00, A380000, A3C2.00, A202.00, A38z.00, A3C0y00, A3Cz.00, K190600, A380300, A380100, L4031HS, SP25400, A3C1y00, A383.00, A384211, A3C.00, Ayu3J00, A3C1z00, Ayu3H00, Ayu3E00, A384100, L090y00, L4034A, A3C0000, A380400, A3C3000, L4033F, L4031, L403100, L4031NM, A3C0z00, L293100, A384400, L4031H, A270600, Ayu3G00, L403.00, L4034AC, A3C2.11, A384000, A3Ay100, A380500, A98yz12, L4031AA, A381100, A383011, A383000, L403000, A38z.11, A3C1000, L40.11, A380.00, A271100, A382.00, A381.00, L090z00, A381000, A38..00, L4031LF, A3C3.00, H5y0100, A384z00, A3C0300, L4031AN, A023.00, A362.00, A3C0100, A384300, L4033FN, A384.00, A396.11, A38y.00, L293.00, A021.00, A270100, A3C3.11, A396.00, A384200, A3C1.00, A3Cy.00, A270611, L4033FA, A3C3y00, L4031LS, Ayu3F00, L4034AB, A3C0.00 | A40.9, A41.2, A41.01, A41.02, A41.1, A40.3, A41.4, A41.50, A41.3, A41.51, A41.52, A41.53, A41.59, A41.89, A41.9, R57.9, R65.20, T81.12XA |
| Urinary tract infection events | K190300, K190.11, 1AG00142512, K190z00, 8CMWE00, K190200, K190500, SP07Q11, tN50.00, K190400, K190.00, K190600 | N390 |
| Gastrointestinal hemorrhage events | J110111, J68z.11 19562, G850.00, J1111111, J121111, G852000, J68.00 13900, J68z000663, J140100, J121300, J110300, intestinal hemorrhage NOS, J11y100, J11yy0010, J10y000, J12yy006, J14y100, J13y100, J13y300, J140300, J130300, J68z.00 597, J68zz00 819, J12y100, J121100, J111300, J131100, J110111, J120300, J120100, J111100 | K922, I8501, I8511, K2211, K2901, K2921, K2931, K2941, K2951, K2961, K2971, K2981, K2991, K31811, K250, K252, K254, K256, K260, K262, K264, K266, K270, K272, K274, K276, K280, K282, K284, K286, K5521, K625, K920 |
| Hypoglycemia events | Q447.00, Cyu3000, C11y100, Q447000, ZV65318, 66A7000, C112100, 66A7100, F374500, J693000, C116.00, C112z00, C116000, C112000, Qyu6000, C112.00, 679L100 | E15, E16.0, E16.1, E16.2 |
| Pancreatitis events | A723.00, A785100, J670.00, J670000, J670100, J670200, J670300, J670400, J670500, J670600, J670700, J670800, J670900, J670y00, J670z00, J670z11, J671.00, J671000, J671100, Jyu8400 | K85.9, K85.90, K85.91, K85.92, K86.1 |
| Select thyroid events | C046.00, C01z.11, C043z00, C051.11, C04z100, C000.13, C03y100, C02yz00, C052.11, C02y000, C050.00, C044.00, C03z.00, C03..00, C04z.12, C01z.00, C03y.00, C022.00, C024z00, C024000, C043200, C05y.00, C011.00, C03z.11, C03z.12, C03..11, C06..00, C041z00, C052.00, C052.12, C062.00, C04z000, C06y000, C03y000, C021000, C061.00, C02z.00, C000.14, C000.12, C020200, C02y100, C050000, C047.00, C0A1.00, C0A3.00, C0A5.00, C06y100, C020.00, C000.11, C025.00, C030.00, C043000, C0.00, C04z.00, C0A.00, C0AX.00, C05z.00, C010.00, C043100, C023z00, C020.12, C063000, C0A4.00, C0A0.00, C023000, C063100, C02.00, C0.11, C00z.00, C045.00, C050200, C05y400, C02zz00, C06yz00, C020100, C06z.00, C05.00, C050z00, C02z100, C041000, C023100, C042.00, C021.00, C023.00, C053.11, C021z00, C043.00, C04.13, C04.12, C04.11, C04z.13, C04z.11, C02.11, C02.12, C00z.11, C040.00, C02y.11, C020000, C02z000, C020.11, C054.00, C022000, C020z00, C06y.00, C02y200, C000.00, C040.11, C01.00, C02y.00, C053.00, C041.00, C04y.00, C060.00, C00.00, C022z00, C02y300, C024.00, C04.00, C051.00, C050100, C031.00 | E04.0, E01.2, E04.9, E04.1, E04.2, E05.00, E05.01, E05.10, E05.11, E05.20, E05.21, E05.30, E05.31, E05.40, E05.80, E05.41, E05.81, E05.90, E05.91, E89.0, E03.2, E01.8, E03.8, E03.9, E06.0, E06.1, E06.3, E06.5, E06.4, E06.9 |
| Acidosis | C10EM00, C101z00, K08y400, C315100, C362.00, C362z00, C101y00, Q448.00, C36A.00, C362200, C362A00, C10A100, K08y900, C10FN11, C101.00, C362900, C362000, C10FN00, C362y00, C10EM11, C362100, C101100, L263.11, C101000, K08yD00 | E87.2 |
| Hyperkalemia | C367.00 | E87.5 |
| Rhabdomyolysis | K04B.00, K045.00, SK09.00, SK08.00, N233300 | M62.82, M60.9, M79.1, M79.7 |
| Select tachycardia events | R050.00, G57y900, 2426.11, G57y700, G570.00, G572z00, 14AQ.00, G571.00, 2426.00, G570000, G571.11, 3282.00, G572.00, R050.12, 14AP.00, G570300, G570z00, G570100, G570200, L263900, G572000, G55A.11 | I47.1, I47.2, I47.9, I48.91, I49.01, I49.40, I49.1, I49.3, I49.49, R00.0, R00.2 |
| Select pneumonia and lower respiratory infection events | H06z011, H06z000, H06z111, H060.00, H06z100, H061.00, H26..00, H062.00, H060.11, H25.00, H21.00, H06.00, H06z200, H260.00, H060z00, H261.00, H060500, H28..00, H060w00, H06z112, H20.00, H2B.00, H260000, H231.00, H22z.00, H061z00, H20z.00, H060300, H22.00, H25.11, H061200, H23.00, H2C.00, H060400, H263.00, H223.00, H060C00, H061500, H06z.00, H22y200, H26.11, H22.11, H224.00, H23z.00, H060600, H20.11, H22yz00, H201.00, H220.00, H262.00, H20y.00, H221.00, H060A00, H24y200, H061100, H240.00, H24y700, H060x00, H233.00, H243.00, H222.00, H24..00, H22y.00, H243.11, H21.11, H060800, H060v00, H061400, H23.11, H24.11, H246.00, H202.00, H24y000, H200.00, H061600, H060000, H060E00, H060D00, H223000, H222.11, H060900, H061300, H060700, H060200, H22y000, H24z.00, H241.00, H247000, H24y.00, H20y000, H242.00, H24y400, H22y011, H061700, H061000, H24y300, H22yX00, H24yz00, H060B00, H232.00, H24y100, H24y600, H060F00, H060100, H22y100, H230.00, H247z00, H203.00, H244.00, H24y500, H247100 | J12.0, J12.1, J12.2, J12.81, J12.89, J12.9, J13, J18.1, J15.0, J15.1, J14, J15.4, J15.3, J15.20, J15.211, J15.212, J15.29, J15.8, J15.5, J15.6, A48.1, J15.9, J15.7, J16.0, J16.8, B25.0, A37.91, A22.1, B44.0, J17, J18.0, J18.9, J11.00 |
| Select hepatic events | J60.00, J600.00, J600000, J600011, J600100, J600200, J600z00, J601.00, J601000, J601100, J601200, J601z00, J60z.00, J61.00, J610.00, J611.00, J612.00, J612.11, J612.12, J612000, J613.00, J613000, J614.00, J614000, J614100, J614111, J614200, J614300, J614400, J614y00, J614z00, J615.00, J615.11, J615100, J615300, J615400, J615500, J615600, J615700, J615711, J615800, J615812, J615C00, J615D00, J615H00, J615y00, J615z00, J615z11, J615z12, J615z13, J615z15, J616.00, J616000, J616100, J616200, J616z00, J617.00, J617000, J61y.00, J61y100, J61y200, J61y300, J61y400, J61y500, J61y600, J61y700, J61y800, J61y900, J61y911, J61yz00, J61z.00, J62.00, J620.00, J620000, J620100, J620200, J620300, J620z00, J621.00, J621.11, J622.00, J622.11, J623.00, J624.00, J625.00, J625.11, J62y.00, J62y.11, J62y.12, J62y.13, J62z.00, J63.00, J630.00, J631.00, J631000, J631100, J631200, J631400, J631500, J631600, J631z00, J632.00, J632000, J632200, J632300, J632z00, J633.00, J633000, J633z00, J634.00, J635.00, J635000, J635100, J635200, J635300, J635400, J635500, J635600, J635700, J635X00, J636.00, J637.00, J638.00, J639.00, J63A.00, J63B.00, J63X.00, J63y.00, J63y000, J63y100, J63y200, J63yz00, J63z.00, J6y.00, J6z.00 | K72.00, K76.2, K71.6, K75.9, K76.1, K76.89, K76.9 |
| Select seizure events | F25.00, 8BIF.00, 667F.00, R003z11, 1473.00, F251000, F251600, 67IJ000, F25z.11, 67AF.00, F254000, 667Q.00, F250000, F25z.00, 282.13, 6672.00, 667P.00, 9N0r.00, 667C.00, 667R.00, 667Z.00, 667S.00, 9Of5.00, F254500, F250011, F255011, F251400, F251200, F251500, F253.11, F25X.00, 667B.00, R003300, 13ZD.00, 667T.00, F132z12, 667X.00, 1O30.00, F256.00, F251300, 6678.00, 667D.00, F251.00, 9Of6.00, 9N4V.00, F25A.00, F25y400, F258.00, SC20000, 1B27.00, F255600, F253.00, 6677.00, 8IAg.00, F250400, 8IAh.00, 667M.00, E201500, 8CE7.00, F255000, 8IAi.00, F252.00, 667V.00, F25y200, 9Of7.00, 9Of4.00, 667A.00, F250200, R003400, F25B.00, F25y300, F254.00, F255.00, F250.00, 9Of3.00, F251011, F25yz00, 6679.00, F25y.00, F25F.00, F251z00, F250500, 667K.00, 667N.00, 6674.00, 667E.00, F254z00, F250300, F25H.00, F256000, F255z00, F256.12, 667W.00, F251100, F251y00, 667G.00, F254100, F256100, 667H.00, F132100, Fyu5100, F250z00, 8Hlp.00, Eu05212, F255y00, F256z00, F254400, Eu80300, F25y500, 667J.00, F259.00, F25y100, F255100, F25C.00, F250y00, F25G.11, F259.11, F251111, F255012, Eu06013, F255200, Fyu5000, F25E.00, F254300, F25D.00, F255300, F254200, F256.11, F250100, F25G.00, F255400, F257.00, F25y000, F255500, F255311, F250 A, F2510 | G40.A01, G40.A09, G40.A11, G40.A19, G40.309, G40.401, G40.409, G40.311, G40.411, G40.419, G40.201, G40.209, G40.211, G40.219, G40.101, G40.109, G40.111, G40.119, G40.501, G40.509, G40.802, G40.804, G40.901, G40.909, G40.911, G40.919, R40.4, R56.9 |
| Select retinal events | F420.00, F420000, F425z00, F41z.00, F425.00, F420400, F425000, F425700, F425.11, F425100, F423800, F42y900, F423600, F420600, F41..00, F421000, F425200, F42y500, F420100, F423811, F425411, F425800, F41.11, F427600, F423.00, F423911, F42..00, F421300, F427z11, F420200, F423z00, F42y.11, F424100, F42z.00, F423900, F423200, F423100, F420z00, F411.00, F421800, F411000, F422011, F421A00, F425412, F426300, F42y.00, F410.00, F421.00, F413100, F425900, F423300, F420300, F426.11, F425400, F426.00, F421F00, F427z12, F42yC00, F424400, F425300, F421900, F427z00, F422.00, F427900, F42yz00, F423211, F42y600, F42y400, F421z00, F427C00, F425600, F42yB00, F421500, F42y700, F422000, F413400, F422z00, F413000, F412.00, F427D00, F421400, F42y000, F425A00, F427800, F423000, F421E00, F426600, F41y000, F421D00, F427.00, F410z00, F413200, F41yz00, F420500, F421H00, F425611, F427300, F413.00, F427J00, F410500, F41y.00, F423400, F426z00, F421A11, F426100, F420700, F422100, F427400, F413z00, F42y100, F427B00, F421700, F424200, F427811, F410000, F425500, F427700, F410600, F411300, F426000, F421.11, F423A00, F420800, F421100, F427G00, F410100, F411z00, F423500, F426400, F423700, F42yA00, F426200, F410800, F410300, F421111, F424300, F411200, F427F00, F410700, F421G00, F427A00, F421600, F427L00, F421112, F411100, F421C00, F427E00, F424.00, F421200, F427y00, F427000, F422y00, F424z00, F421B00, F413300, F410400, F426500, F42y300, F427K00, F427500, F410200, F42yD00, F427H00, F427L11, F411500, F410900, F424000, F42y800, F425211, F411400, F427K11, F427200, F426311, F426211, F4109TC, F4139N, F4129AN, F4109N, F4290D | E11.311, E11.319, E11.36, E11.39, E10.311, E10.319, E10.36, E10.37X1, E10.37X2, E10.37X3, E10.37X9, E10.39, E11.65, E10.65, E11.359, E11.3591, E11.3592, E11.3593, E11.3599, E11.329, E11.3291, E11.3292, E11.3293, E11.3299, E11.349, E11.3491, E11.3492, E11.3493, E11.3499, H34.8110, H34.8111, H34.8112, H34.8120, H34.8121, H34.8122, H34.8130, H34.8131, H34.8132, H34.819, H34.8190, H34.8191, H34.8192, H35.60, H35.81, H35.82 |
| Select allergic and anaphylaxis events | SN50.00, F401400, 1Z30.00, 1Z3.00 | T78.2XXA, T78.3XXA, T50.995A |
| Severe cutaneous adverse reactions | N/A | L51.0, L51.9, L51.8, L51.1, L51.3, L51.2, L49.0, L49.1, L49.2, L49.3, L49.4, L49.5, L49.6, L49.7, L49.8, L49.9 |
| Pure red cell aplasia | D201800, D200312, D20X.00, D202.00, D203.00 | D61.01 |
| Pyelonephritis | A0225, D8684 | N10, N110, N111 |

*Abbreviations:* ICD-10, International Classification of Diseases, Tenth Revision; N/A, not applicable.

Table S2 Patient mortality and risk by dialysis status and modality

| **Patient mortality and risk** | | **DD-CKD** | | | **IDD-CKD** | | |
| --- | --- | --- | --- | --- | --- | --- | --- |
|  |  | **HD**  **(*n* = 2,679)** | **PD**  **(*n* = 776)** | **NS**  **(*n* = 476)** | **HD**  **(*n* = 2,220)** | **PD**  **(*n* = 699)** | **NS**  **(*n* = 411)** |
| 1 year | Events, *n* | 627 | 88 | 142 | 619 | 87 | 139 |
|  | Mortality risk, KM% (95% CI) | 24.09 (22.43–25.72) | 11.76 (9.42–14.05) | 30.91 (26.53–35.03) | 28.87 (26.92–30.77) | 12.95 (10.37–15.46) | 35.23 (30.31–39.80) |
| 2 years | Events, *n* | 212 | 49 | 38 | 193 | 48 | 36 |
|  | Mortality risk, KM% (95% CI) | 33.47 (31.58–35.31) | 19.12 (16.17–21.96) | 40.53 (35.71–44.98) | 39.56 (37.37–41.67) | 21.08 (17.83–24.20) | 46.21 (40.84–51.10) |
| 3 years | Events, *n* | 145 | 50 | 23 | 118 | 46 | 22 |
|  | Mortality risk, KM% (95% CI) | 40.83 (38.79–42.81) | 27.65 (24.14–31.00) | 47.13 (42.06–51.75) | 47.25 (44.92–49.49) | 30.06 (26.22–33.69) | 54.00 (48.36–59.03) |
| 5 years | Events, *n* | 222 | 76 | 18 | 176 | 66 | 13 |
|  | Mortality risk, KM% (95% CI) | 54.89 (52.59–57.08) | 43.83 (39.47–47.87) | 53.48 (48.11–58.30) | 61.94 (59.35–64.36) | 46.43 (41.71–50.77) | 59.95 (54.00–65.13) |

Where KM% is KM risk function multiplied by 100, including estimated 95% CI.

*Abbreviations:* CI, confidence interval; CKD, chronic kidney disease; DD, dialysis-dependent; HD, hemodialysis; IDD, incident dialysis-dependent; KM, Kaplan–Meier; NS, dialysis modality not specified; PD, peritoneal dialysis.


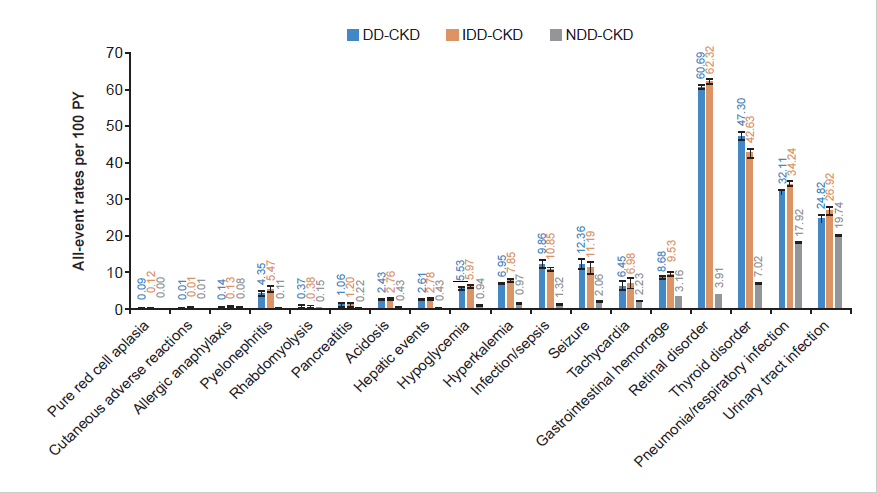
Fig. S1 All-event rates per 100 PY (95% CI) for patients with CKD by dialysis status.

*Abbreviations:* CI, confidence interval; CKD, chronic kidney disease; DD, dialysis-dependent; IDD, incident dialysis-dependent; NDD, non–dialysis-dependent; PY, person-years.

Fig. S2 Incidence rates of adverse clinical events per 100 PY (95% CI) for patients with CKD over time.

1.
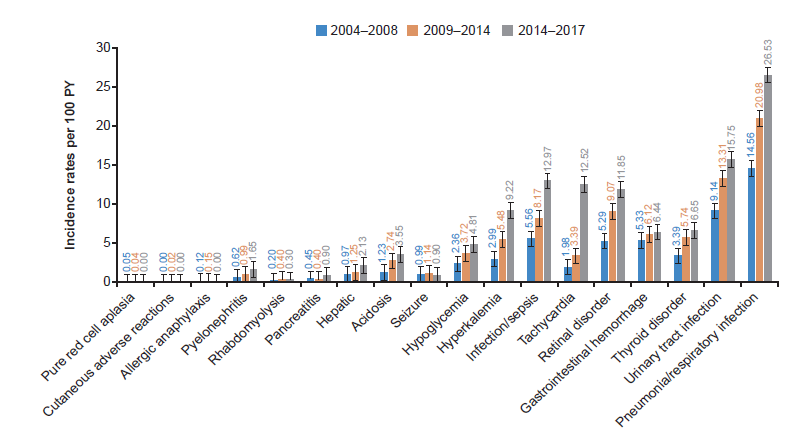
**DD-CKD**
2.
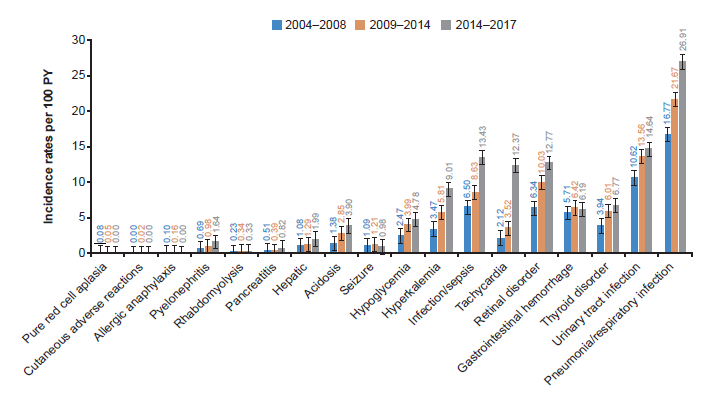
**IDD-CKD**
3.
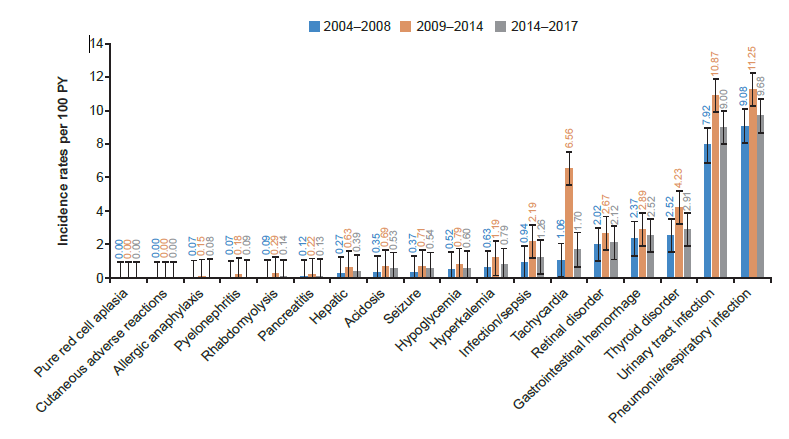
**NDD-CKD**

*Abbreviations:* CI, confidence interval; CKD; chronic kidney disease; DD, dialysis-dependent; IDD, incident dialysis-dependent; NDD, non–dialysis-dependent; PY, person-years.

Fig. S3 All-event rates per 100 PY (95% CI) for patients with NDD-CKD by disease stage.

**
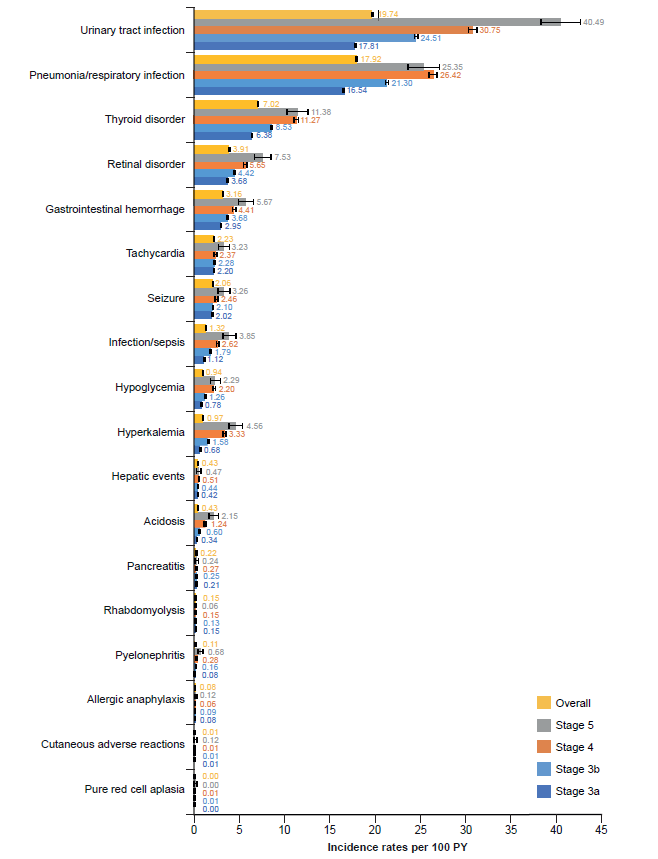
**

*Abbreviations:* CI, confidence interval; CKD, chronic kidney disease; NDD, non–dialysis-dependent; PY, person-years.

Fig. S4 Incidence rates per 100 PY (95% CI) for patients with DD-CKD or IDD-CKD by dialysis modality.

1.
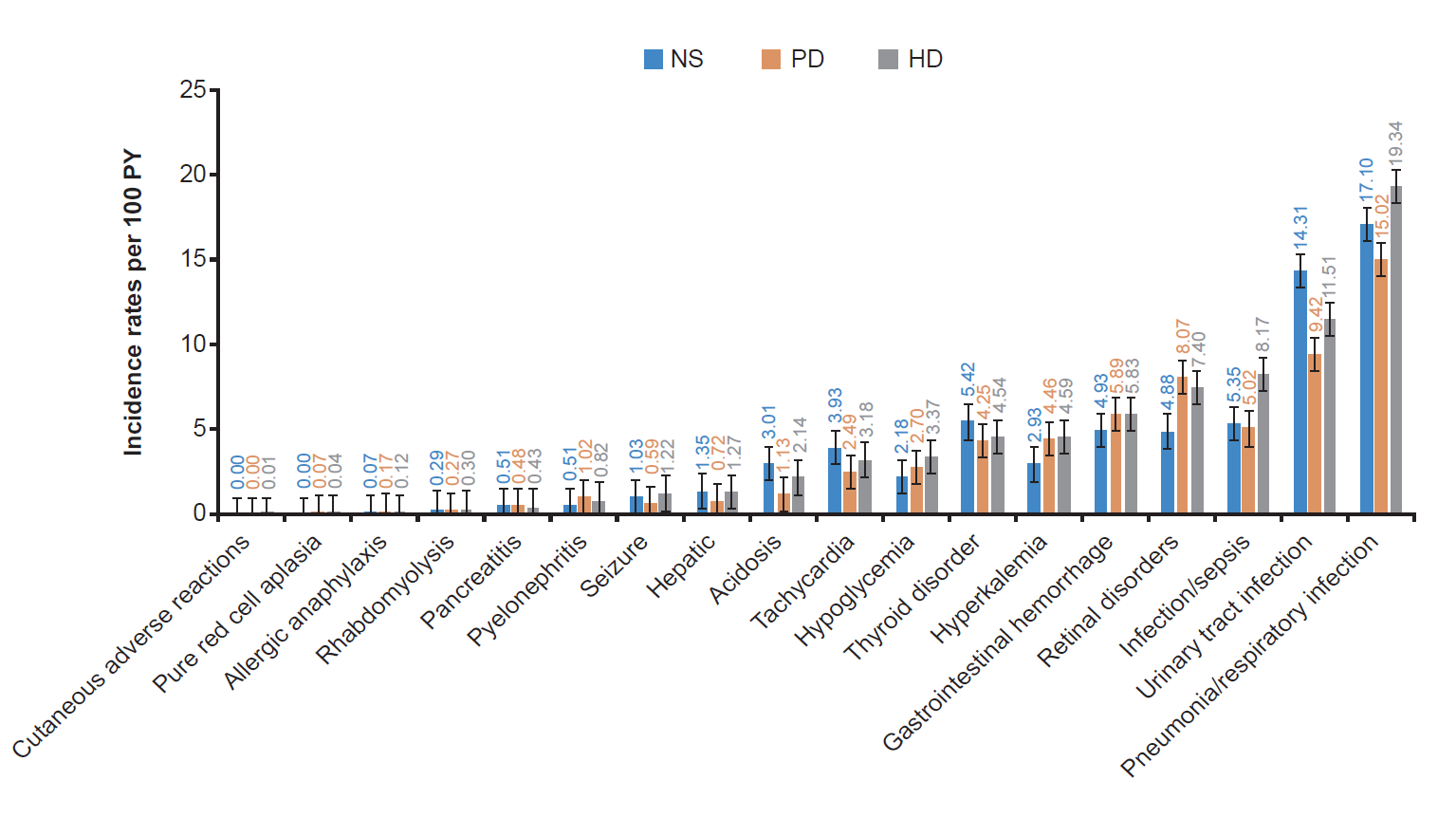
**DD-CKD**
2.
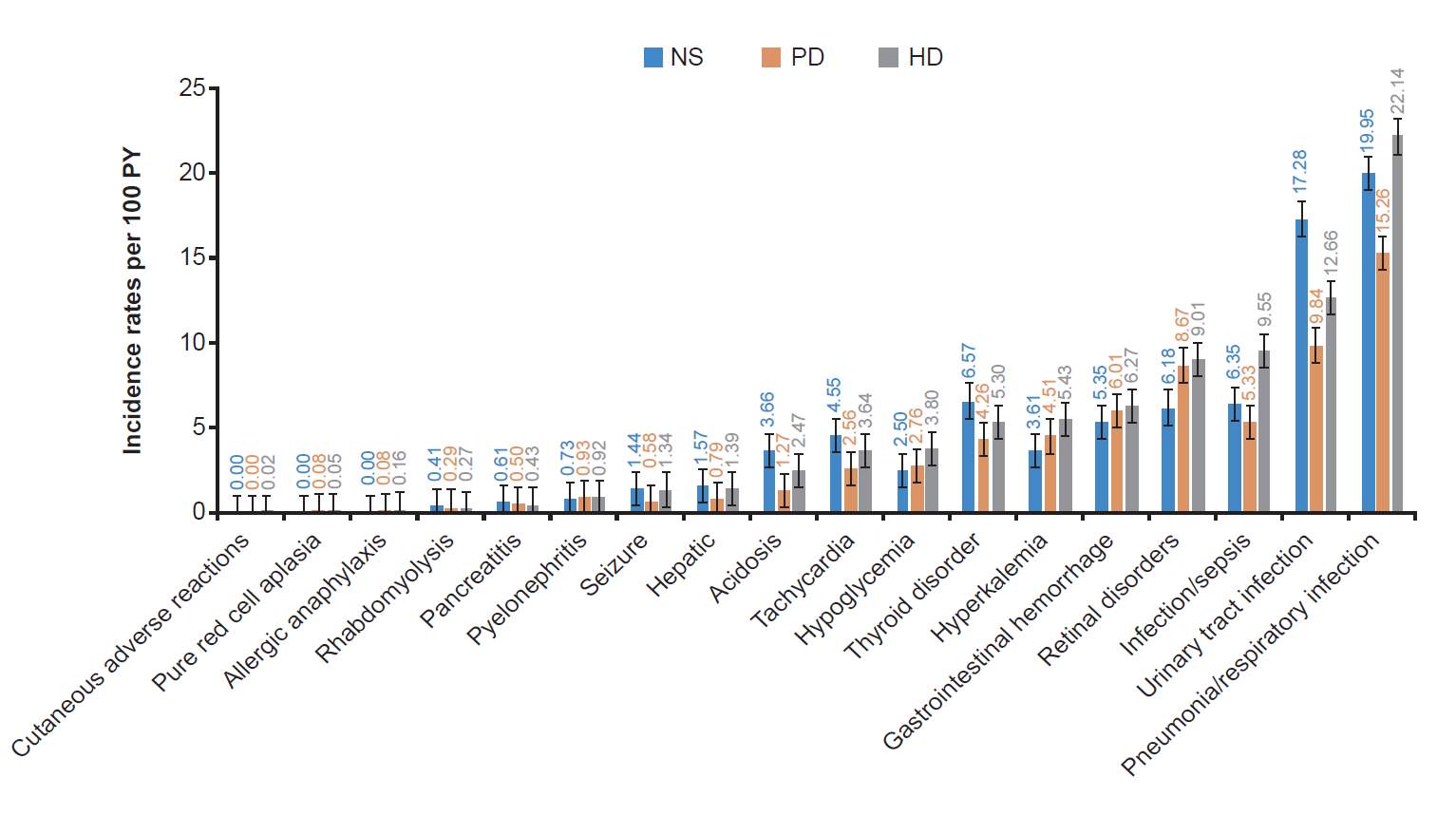
**IDD-CKD**

*Abbreviations:* CI, confidence interval; CKD, chronic kidney disease; DD, dialysis-dependent; HD, hemodialysis; IDD, incident dialysis-dependent; NS, dialysis modality not specified; PD, peritoneal dialysis; PY, person-years.

Fig. S5 Risk of mortality for patients with NDD-CKD by stage, IDD-CKD, and DD-CKD.


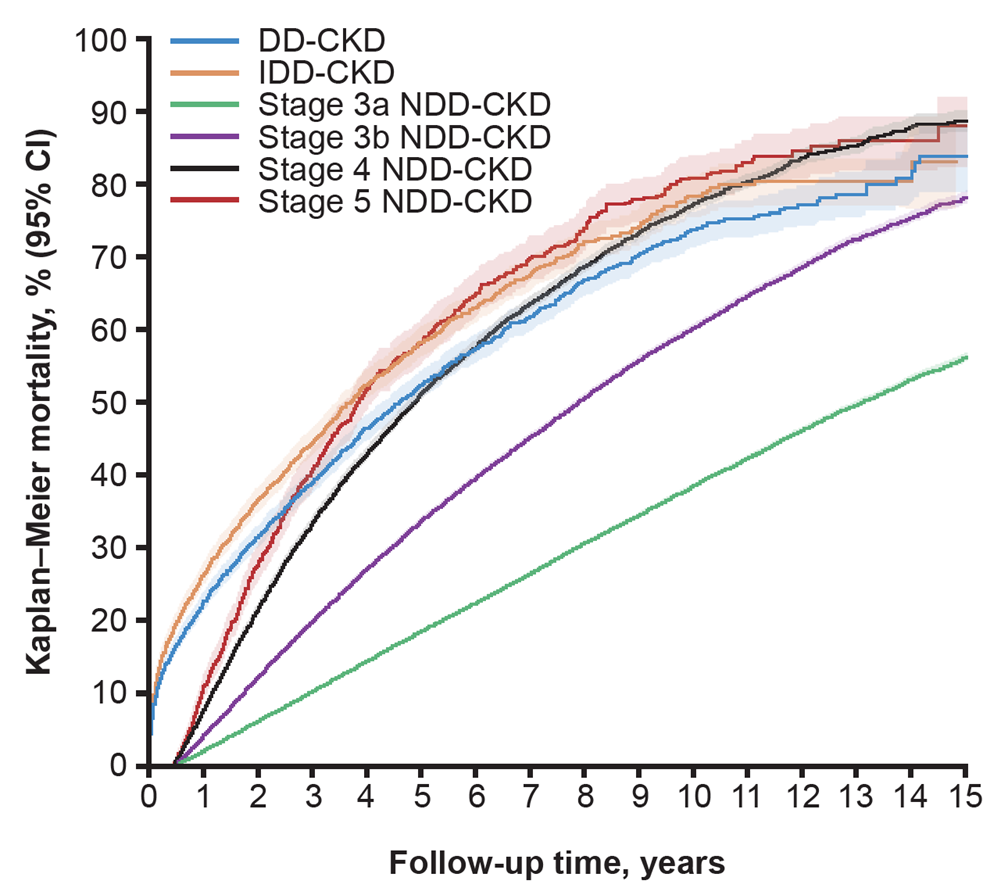


Shaded area represents 95% CIs of Kaplan–Meier mortality risk (%).

*Abbreviations:* CI, confidence interval; CKD, chronic kidney disease; DD, dialysis-dependent; IDD, incident dialysis-dependent; NDD, non–dialysis-dependent.
